# Supplementary material for: Effect of disinfection agents and quantification of potentially viable Leptospira in fresh water samples using a highly sensitive integrity-qPCR assay
Source: PLoS One. 2021 May 26;16(5):e0251901. doi: 10.1371/journal.pone.0251901 (PMC8153454; doi:10.1371/journal.pone.0251901)
Supplement: S1 Table — (DOCX) [file pone.0251901.s001.docx]

S1 Table. Microorganism strains used for specificity tests and results from the TaqMan real-time multiplex (LipL32 and 16S) PCR assays.

| **Gender** | **Status** | **Species** | **Results PCR 16S-*Leptospira*** | **Results PCR LipL32-*Leptospira*** |
| --- | --- | --- | --- | --- |
|  | Saprophyte S1 | *L. biflexa* | + | - |
|  | Saprophyte S1 | *L. terpstrae* | + | - |
|  | Saprophyte S1 | *L. vanthielii* | + | - |
|  | Saprophyte S1 | *L. yanagawae* | + | - |
|  | Saprophyte S2 | *L. kobayashi* | + | - |
|  | Saprophyte S2 | *L. idonii* | + | - |
|  | Saprophyte S2 | *L. ognonensis* | + | - |
|  | Saprophyte S2 | *L. ryugenii* | + | - |
|  | Pathogen P1 | *L. adleri* | + | ND |
|  | Pathogen P1 | *L. alstonii* | + | + |
|  | Pathogen P1 | *L. barantonii* | + | ND |
|  | Pathogen P1 | *L. borgpetersenii* | + | + |
|  | Pathogen P1 | *L. ellisii* | + | + |
|  | Pathogen P1 | *L. interrogans* | + | + |
|  | Pathogen P1 | *L. kirschnerii* | + | + |
|  | Pathogen P1 | *L. kmetyi* | + | ND |
|  | Pathogen P1 | *L. mayottensis* | + | ND |
|  | Pathogen P1 | *L. noguchii* | + | + |
|  | Pathogen P1 | *L. santarosai* | + | + |
|  | Pathogen P1 | *L. weilli* | + | + |
|  | Pathogen P2 | *L. fainei* | + | - |
|  | Pathogen P2 | *L. johnsonii* | + | - |
|  | Pathogen P2 | *L. licerasiae* | + | - |
|  | Pathogen P2 | *L. venezuelensis* | + | - |
|  | Pathogen P2 | *L. wolffii* | + | - |
| *Enterococcus* |  | *E. faecalis* | - | NT |
| *Escherichia* |  | *E. coli* | - | NT |
| *Klebsiella* |  | *K. pneumoniae* | - | NT |
| *Pseudomonas* |  | *P. aeruginosa* | - | NT |
| *Salmonella* |  | *S. enterica* | - | NT |
| *Staphylococcus* |  | *Spp* | - | NT |

The two PCR assays tested were specific for *Leptospira*. *LipL32* was specific for pathogenic *Leptospira* species belonging to the P1 group. ND: not determined, NT: not tested. +: positive PCR output, -: no PCR signal
