# Supplementary material for: Effect of disinfection agents and quantification of potentially viable Leptospira in fresh water samples using a highly sensitive integrity-qPCR assay
Source: PLoS One. 2021 May 26;16(5):e0251901. doi: 10.1371/journal.pone.0251901 (PMC8153454; doi:10.1371/journal.pone.0251901)
Supplement: S2 Table — (DOCX) [file pone.0251901.s002.docx]

S2 Table. Leptospira sequences and their NCBI accession numbers required to design primers and probe for 16S qPCR (www.ncbi.nlm.nih.gov/)

| **No** | **Organism** |  | **Serovar** | **Group** | **Accession N°** |  |
| --- | --- | --- | --- | --- | --- | --- |
| 1 | *Leptospira* | *alexanderi* | Manhao | Pathogenic | AY631880 |  |
| 2 | *Leptospira* | *alexanderi* | Manhao 3 | Pathogenic | NR_043047 |  |
| 3 | *Leptospira* | *alexanderi* | Manzhuang | Pathogenic | AY996803 |  |
| 4 | *Leptospira* | *alexanderi* | Mengla | Pathogenic | DQ991481 |  |
| 5 | *Leptospira* | *alexanderi* | Nanding | Pathogenic | AY996804 |  |
| 6 | *Leptospira* | *alstonii* | Pingchang | Pathogenic | AOHD02000026 |  |
| 7 | *Leptospira* | *alstonii* | Pingchang | Pathogenic | DQ991480 |  |
| 8 | *Leptospira* | *alstonii* | Sichuan | Pathogenic | AY631881 |  |
| 9 | *Leptospira* | *borgpetersenii* | Balcanica | Pathogenic | U12669 |  |
| 10 | *Leptospira* | *borgpetersenii* | Ballum | Pathogenic | AM050581 |  |
| 11 | *Leptospira* | *borgpetersenii* | Ballum | Pathogenic | JQ988847 |  |
| 12 | *Leptospira* | *borgpetersenii* | Ballum | Pathogenic | FJ154591 |  |
| 13 | *Leptospira* | *borgpetersenii* | Ballum | Pathogenic | KU219452 |  |
| 14 | *Leptospira* | *borgpetersenii* | Ceylonica | Pathogenic | FJ154596 |  |
| 15 | *Leptospira* | *borgpetersenii* | Hardjo | Pathogenic | FJ154586 |  |
| 16 | *Leptospira* | *borgpetersenii* | Hardjo | Pathogenic | KU219450 |  |
| 17 | *Leptospira* | *borgpetersenii* | Javanica | Pathogenic | AY887899 |  |
| 18 | *Leptospira* | *borgpetersenii* | Javanica | Pathogenic | NZ_AOUW01000091 |  |
| 19 | *Leptospira* | *borgpetersenii* | Sejroe | Pathogenic | JQ988862 |  |
| 20 | *Leptospira* | *borgpetersenii* | Sejroe | Pathogenic | KR107201 |  |
| 21 | *Leptospira* | *borgpetersenii* | Sejroe | Pathogenic | FJ154593 |  |
| 22 | *Leptospira* | *borgpetersenii* | Tarassovi | Pathogenic | JQ988861 |  |
| 23 | *Leptospira* | *interrogans* | Australis | Pathogenic | AY996794 |  |
| 24 | *Leptospira* | *interrogans* | Australis | Pathogenic | JQ988863 |  |
| 25 | *Leptospira* | *interrogans* | Australis | Pathogenic | FJ154557 |  |
| 26 | *Leptospira* | *interrogans* | Autumnalis | Pathogenic | AY996791 |  |
| 27 | *Leptospira* | *interrogans* | Bratislava | Pathogenic | JQ988859 |  |
| 28 | *Leptospira* | *interrogans* | Bratislava | Pathogenic | AM050583 |  |
| 29 | *Leptospira* | *interrogans* | Bataviae | Pathogenic | EF536987 |  |
| 30 | *Leptospira* | *interrogans* | Bataviae | Pathogenic | AM050582 |  |
| 31 | *Leptospira* | *interrogans* | Bataviae | Pathogenic | FJ154566 |  |
| 32 | *Leptospira* | *interrogans* | Bulgarica | Pathogenic | AY996792 |  |
| 33 | *Leptospira* | *interrogans* | Canicola | Pathogenic | KR080516 |  |
| 34 | *Leptospira* | *interrogans* | Canicola | Pathogenic | AM050566 |  |
| 35 | *Leptospira* | *interrogans* | Canicola | Pathogenic | KU053945 |  |
| 36 | *Leptospira* | *interrogans* | Copenhageni | Pathogenic | GQ204292 |  |
| 37 | *Leptospira* | *interrogans* | Copenhageni | Pathogenic | KY000352 |  |
| 38 | *Leptospira* | *interrogans* | Copenhageni | Pathogenic | KR030154 |  |
| 39 | *Leptospira* | *interrogans* | Copenhageni | Pathogenic | FJ154569 |  |
| 40 | *Leptospira* | *interrogans* | Djasiman | Pathogenic | FJ154550 |  |
| 41 | *Leptospira* | *interrogans* | Fortbragg | Pathogenic | JQ906634 |  |
| 42 | *Leptospira* | *interrogans* | Grippotyphosa | Pathogenic | JQ906696 |  |
| 43 | *Leptospira* | *interrogans* | Grippotyphosa | Pathogenic | JQ906628 |  |
| 44 | *Leptospira* | *interrogans* | Hardjo | Pathogenic | AM050568 |  |
| 45 | *Leptospira* | *interrogans* | Hardjo | Pathogenic | AY996797 |  |
| 46 | *Leptospira* | *interrogans* | Hebdomadis | Pathogenic | JQ988848 |  |
| 47 | *Leptospira* | *interrogans* | Hebdomadis | Pathogenic | AM050570 |  |
| 48 | *Leptospira* | *interrogans* | Hebdomadis | Pathogenic | JQ906633 |  |
| 49 | *Leptospira* | *interrogans* | Icterohaemorrhagiae | Pathogenic | AY631894 |  |
| 50 | *Leptospira* | *interrogans* | Icterohaemorrhagiae | Pathogenic | JQ988845 |  |
| 51 | *Leptospira* | *interrogans* | Icterohaemorrhagiae | Pathogenic | FJ154555 |  |
| 52 | *Leptospira* | *interrogans* | Icterohaemorrhagiae | Pathogenic | FJ154563 |  |
| 53 | *Leptospira* | *interrogans* | Icterohaemorrhagiae | Pathogenic | KU053947 |  |
| 54 | *Leptospira* | *interrogans* | Javanica | Pathogenic | KY075910 |  |
| 55 | *Leptospira* | *interrogans* | Kennewicki | Pathogenic | FJ154571 |  |
| 56 | *Leptospira* | *interrogans* | Kirikkale | Pathogenic | HM536967 |  |
| 57 | *Leptospira* | *interrogans* | Kirikkale | Pathogenic | HM536965 |  |
| 58 | *Leptospira* | *interrogans* | Kremastos | Pathogenic | DQ991467 |  |
| 59 | *Leptospira* | *interrogans* | Linhai | Pathogenic | JQ906636 |  |
| 60 | *Leptospira* | *interrogans* | Manilae | Pathogenic | FJ154545 |  |
| 61 | *Leptospira* | *interrogans* | Parameles | Pathogenic | AB368965 |  |
| 62 | *Leptospira* | *interrogans* | Pomona | Pathogenic | JQ988858 |  |
| 63 | *Leptospira* | *kirschneri* | Agogo | Pathogenic | DQ991476 |  |
| 64 | *Leptospira* | *kirschneri* | Grippotyphosa | Pathogenic | FJ154572 |  |
| 65 | *Leptospira* | *kirschneri* | Grippotyphosa | Pathogenic | JQ988856 |  |
| 66 | *Leptospira* | *kirschneri* | Mozdoc | Pathogenic | AM050574 |  |
| 67 | *Leptospira* | *kirschneri* | Cynopteri | Pathogenic | AY631895 |  |
| 68 | *Leptospira* | *kmetyi* | Malaysia | Pathogenic | AHMP02000003 |  |
| 69 | *Leptospira* | *kmetyi* | Malaysia | Pathogenic | NR_041544 |  |
| 70 | *Leptospira* | *mayottensis* | Not determined | Pathogenic | KJ847187 |  |
| 71 | *Leptospira* | *mayottensis* | Not determined | Pathogenic | KT338879 |  |
| 72 | *Leptospira* | *mayottensis* | Not determined | Pathogenic | JN683863 |  |
| 73 | *Leptospira* | *noguchii* | Autumnalis | Pathogenic | AHOP02000022 |  |
| 74 | *Leptospira* | *noguchii* | Panama | Pathogenic | NR_043050 |  |
| 75 | *Leptospira* | *santarosai* | Not determined | Pathogenic | AY461889 |  |
| 76 | *Leptospira* | *santarosai* | Not determined | Pathogenic | U12672 |  |
| 77 | *Leptospira* | *santarosai* | Shermani | Pathogenic | AY631883 |  |
| 78 | *Leptospira* | *santarosai* | Bananal | Pathogenic | KJ946437 |  |
| 79 | *Leptospira* | *weilii* | Celledoni | Pathogenic | AY631877 |  |
| 80 | *Leptospira* | *broomii* | Not determined | Intermediate | AY792329 |  |
| 81 | *Leptospira* | *broomii* | Not determined | Intermediate | AY796065 |  |
| 82 | *Leptospira* | *broomii* | Not determined | Intermediate | Y19243 |  |
| 83 | *Leptospira* | *fainei* | Hurstbridge | Intermediate | AY996789 |  |
| 84 | *Leptospira* | *fainei* | Hurstbridge | Intermediate | FJ154578 |  |
| 85 | *Leptospira* | *inadai* | Aguaruna | Intermediate | AY631891 |  |
| 86 | *Leptospira* | *inadai* | Kaup | Intermediate | AY631887 |  |
| 87 | *Leptospira* | *inadai* | Lyme | Intermediate | AY631896 |  |
| 88 | *Leptospira* | *inadai* | Lyme | Intermediate | JQ988844 |  |
| 89 | *Leptospira* | *licerasiae* | Varillal | Intermediate | EF612278 |  |
| 90 | *Leptospira* | *licerasiae* | Not determined | Intermediate | IP |  |
| 91 | *Leptospira* | *wolffii* | Khorat | Intermediate | EF025496 |  |
| 92 | *Leptospira* | *wolffii* | Khorat | Intermediate | NR_044042 |  |
| 93 | *Leptospira* | *biflexa* | Ancona | Saprophytic | Z21629 |  |
| 94 | *Leptospira* | *biflexa* | Andamana | Saprophytic | AY631893 | |
| 95 | *Leptospira* | *biflexa* | Canela | Saprophytic | Z21631 | |
| 96 | *Leptospira* | *biflexa* | Jequitaia | Saprophytic | Z21633 | |
| 97 | *Leptospira* | *biflexa* | Patoc | Saprophytic | AY631876 | |
| 98 | *Leptospira* | *biflexa* | Patoc | Saprophytic | NR_043043 | |
| 99 | *Leptospira* | *biflexa* | Patoc | Saprophytic | IP | |
| 100 | *Leptospira* | *biflexa* | Patoc | Saprophytic | AF157070 | |
| 101 | *Leptospira* | *idonii* | Not determined | Saprophytic | AB721966 | |
| 102 | *Leptospira* | *idonii* | Not determined | Saprophytic | NR_114336 | |
| 103 | *Leptospira* | *meyeri* | Hardjo | Saprophytic | AY631889 | |
| 104 | *Leptospira* | *meyeri* | Semaranga | Saprophytic | AY631892 | |
| 105 | *Leptospira* | *meyeri* | Ranarum | Saprophytic | AY631878 | |
| 106 | *Leptospira* | *terpstrae* | Hualin | Saprophytic | AY631888 | |
| 107 | *Leptospira* | *terpstrae* | Hualin | Saprophytic | NR_115294 | |
| 108 | *Leptospira* | *vanthielii* | Holland | Saprophytic | AY631897 | |
| 109 | *Leptospira* | *vanthielii* | Holland | Saprophytic | NR_115297 | |
| 110 | *Leptospira* | *wolbachii* | Codice | Saprophytic | AY631879 | |
| 111 | *Leptospira* | *wolbachii* | Codice | Saprophytic | NR_043046 | |
| 112 | *Leptospira* | *yanagawae* | Saopaulo | Saprophytic | AY631882 | |
| 113 | *Leptospira* | *yanagawae* | Saopaulo | Saprophytic | NR_115293 | |
