# Supplementary material for: Effect of disinfection agents and quantification of potentially viable Leptospira in fresh water samples using a highly sensitive integrity-qPCR assay
Source: PLoS One. 2021 May 26;16(5):e0251901. doi: 10.1371/journal.pone.0251901 (PMC8153454; doi:10.1371/journal.pone.0251901)
Supplement: S3 Table — (DOCX) [file pone.0251901.s003.docx]

S3 Table. Leptospira sequences and their NCBI accession numbers required to design primers and probe for the LipL32 qPCR

| **No** | **Organism** | | **Serovar** | **Group** | **Accession number** |
| --- | --- | --- | --- | --- | --- |
| 1 | *Leptospira* | *alstonii* | Pingchang | Pathogenic | NZ_AOHD02000048 |
| 2 | *Leptospira* | *alstonii* | Sichuan | Pathogenic | NZ_ANIK01000029 |
| 3 | *Leptospira* | *borgpetersenii* | Ballum | Pathogenic | KU219466 |
| 4 | *Leptospira* | *borgpetersenii* | Ballum | Pathogenic | AY609322 |
| 5 | *Leptospira* | *borgpetersenii* | Hardjo | Pathogenic | KU219468 |
| 6 | *Leptospira* | *borgpetersenii* | Mini | Pathogenic | AY609333 |
| 7 | *Leptospira* | *interrogans* | Australis | Pathogenic | AY609325 |
| 8 | *Leptospira* | *interrogans* | Autumnalis | Pathogenic | AY609324 |
| 9 | *Leptospira* | *interrogans* | Autumnalis | Pathogenic | JN210551 |
| 10 | *Leptospira* | *interrogans* | Balico | Pathogenic | JQ013520 |
| 11 | *Leptospira* | *interrogans* | Canicola | Pathogenic | AY609321 |
| 12 | *Leptospira* | *interrogans* | Canicola | Pathogenic | KC800990 |
| 13 | *Leptospira* | *interrogans* | Copenhageni | Pathogenic | GQ204288 |
| 14 | *Leptospira* | *interrogans* | Copenhageni | Pathogenic | GQ204287 |
| 15 | *Leptospira* | *interrogans* | Copenhageni | Pathogenic | KU219470 |
| 16 | *Leptospira* | *interrogans* | Grippotyphosa | Pathogenic | EU871723 |
| 17 | *Leptospira* | *interrogans* | Grippotyphosa | Pathogenic | JN886738 |
| 18 | *Leptospira* | *interrogans* | Hardjo | Pathogenic | JN886739 |
| 19 | *Leptospira* | *interrogans* | Hardjo | Pathogenic | KC800991 |
| 20 | *Leptospira* | *interrogans* | Hebdomadis | Pathogenic | GU220823 |
| 21 | *Leptospira* | *interrogans* | Hebdomadis | Pathogenic | AB094436 |
| 22 | *Leptospira* | *interrogans* | Hebdomadis | Pathogenic | AY609328 |
| 23 | *Leptospira* | *interrogans* | Icterohaemorrhagiae | Pathogenic | AB094433 |
| 24 | *Leptospira* | *interrogans* | Icterohaemorrhagiae | Pathogenic | KC800993 |
| 25 | *Leptospira* | *interrogans* | Javanica | Pathogenic | AY776292 |
| 26 | *Leptospira* | *interrogans* | Kremastos | Pathogenic | AY461906 |
| 27 | *Leptospira* | *interrogans* | Manilae | Pathogenic | JQ013519 |
| 28 | *Leptospira* | *kirschneri* | Not determined | Pathogenic | AY461917 |
| 29 | *Leptospira* | *kirschneri* | Not determined | Pathogenic | AY461916 |
| 30 | *Leptospira* | *kmetyi* | Malaysia | Pathogenic | NZ_AHMP02000003 |
