# Supplementary material for: Effect of disinfection agents and quantification of potentially viable Leptospira in fresh water samples using a highly sensitive integrity-qPCR assay
Source: PLoS One. 2021 May 26;16(5):e0251901. doi: 10.1371/journal.pone.0251901 (PMC8153454; doi:10.1371/journal.pone.0251901)
Supplement: Attachment — Submitted filename: Reviewer PONE-D-09857.docx [file pone.0251901.s004.docx]

### UNIVERSIDADE FEDERAL DE PELOTAS

FACULDADE DE VETERINÁRIA

DEPARTAMENTO DE CLÍNICAS VETERINÁRIAS

Apr, 2021

PONE-D-21-09857

The Editor Odir Dellagostin

PLOS ONE

The manuscript entitled "Effect of disinfection agents on Leptospira in water using a high sensitivity integrity qPCR assay” bring a new quantitative polymerase chain reaction coupled to propidium monoazide treatment (PMAqPCR) for detection of *Leptospira* spp. in treated water. The work is interesting and well written. However some minor revisions are needed before the manuscript is considered for publication.

**Minor points:**

**Title**

I suggest that the title be rewritten. The evaluated method showed limitations when used in treated water; however it showed good results for analyzing fresh water**.**

**Introduction**

P.4, L.16 – Include “(CFR)” behind “Case Fatality Rate”;

P.4, L.36 – Change Clean and Health Lifestyle for lowercase letters;

P.4, L. 47 – Include in sentence “….to risk factors in community behavior..”.

**Matherial and methods**

Include geographic coordinates;

P.5, L. 3 - What laboratory tests were performed?;

P.5 L.7 - The clinical diagnosis is presumptive and must be confirmed at the laboratory diagnosis, this point must be clarified;

P.5, L. 28 - How bathing habits can affect leptospirosis incidence?;

P.5, L.29 – The leptospirosis transmission from rats to humans is frequently through indirect contact with animal urine contaminated, so, why were evaluated direct transmission by contact with rat carcasses?;

P.5, L.32 - Statistical analyzes should be more detailed. Include the software that was used in the analysis.

**Results**

Why were all the years studied (20116-2019) not included in tables 1 and 2?

**Discussion**

P.7, L.42 – Include references.

**References**

The formatting of references must be updated.

Sincerely,

Dr. Sérgio Jorge

Universidade Federal de Pelotas

Faculdade de Veterinária

Email: sergiojorgevet@hotmail.com
