# Supplementary material for: Effect of disinfection agents and quantification of potentially viable Leptospira in fresh water samples using a highly sensitive integrity-qPCR assay
Source: PLoS One. 2021 May 26;16(5):e0251901. doi: 10.1371/journal.pone.0251901 (PMC8153454; doi:10.1371/journal.pone.0251901)
Supplement: Attachment — Submitted filename: RefereeReplyfinal.docx [file pone.0251901.s005.docx]

PONE-D-21-09857

Dear Editor,

Thanks for considering our work to be published in your journal. We replied to all the referee remarks and your comments below, in blue.

Sincerely,

Laurent Moulin

Reviewer #1: I suggest that the title be rewritten. The evaluated method showed limitations when used in treated water; however it showed good results for analyzing fresh water.
As suggested, the title was rewritten.

Introduction
P.3, L.36 – Include references;
References added as requested.

P.4, L.64 – Amphotericin B is an antifungal, correct in the text;
Corrected as suggested

P.4, L.65 – Include dark field microscopy in the sentence;
Dark field microscopy has been included in the sentence.

P.5, L.84 – Include references.
References added as requested.

Matherial and methods
P. 12, L.229 - Include geographic coordinates;
Geographical coordinates were added for samples from the Seine river and from the bathing area.

Results
P.13, L.243 – 248 - Remove this paragraph, this information has already been given.
This paragraph was removed as suggested.

Discussion
P.21, L.404 – The authors the authors identified that the pathogenic strain being more tolerant to heat. This is an interesting finding and should be discussed further.
As suggested, this result is now discussed.

Reviewer #2:

The present study is well-written and properly conducted. The Discussion is the section that stands out with an interesting debate about the possible uses of the technique with an impact on public health. I am not sure of the speed of the developed method, so I suggest modify the objective to: investigate the accuracy of a new molecular method for the quantification of potentially viable leptospires in environmental water samples under disinfectants influence.

Many thanks to the reviewers for these nice comments

Minor revisions:
L63-68; L83-84: are not required in the Introduction.

Lanes 63-68 has been shortened and Lanes 83-84 has been removed, as suggested

L99 (We implemented ....) - 105 (... animal samples) must be moved to the beginning of the Discussion.
As suggested, this part has been moved to the beginning of the discussion.

Do not give paragraph on line 96
Paragraph line 96 has been removed

In Material and Methods, specify the water collection points better. How many points in the Paris city were collected? Did all points receive water treatment included in the city's sanitation?
Geographical localization and number of samples were added to this paragraph. These samples were composed by surface water which had not undergone any sanitation treatment.

L371-379: remove from Discussion. This paragraph is not part of the debate.

Most of the paragraph has been shortened
